# Establishing a Surgical Procedure for Rhesus Epiretinal Scaffold Implantation with HiPSC-Derived Retinal Progenitors

**DOI:** 10.1155/2018/9437041

**Published:** 2018-04-15

**Authors:** Ziming Luo, Kang Li, Kaijing Li, Bikun Xian, Ying Liu, Sijing Yang, Chaochao Xu, Zhigang Fan, Shoutao Lu, Haijun Zhang, Jian Ge

**Affiliations:** ^1^State Key Laboratory of Ophthalmology, Zhongshan Ophthalmic Center, Sun Yat-sen University, Guangzhou, Guangdong 510060, China; ^2^Bai Duoan Medical Equipment Company, Dezhou, Shandong 251100, China

## Abstract

**Background:**

To develop an effective surgical procedure for cellular scaffold epiretinal implantation in rhesus, facilitating subsequent epiretinal stem cell transplantation.

**Methods:**

Retinal progenitors were seeded onto a poly(lactic-co-glycolic) acid (PLGA) scaffold. First, the cellular scaffolds were delivered by 18G catheter or retinal forceps into rabbit epiretinal space (*n* = 50). Then, the cell survival rate was evaluated by Cell Counting Kit-8 (CCK-8). Second, three methods of scaffold fixation, including adhesion after gas-liquid exchange (*n* = 1), tamponade by hydrogel (*n* = 1), and fixation by retinal tacks (*n* = 4), were performed in rhesus monkeys. After one month, fundus photography and SD-OCT were performed to assess the outcomes, and histological examination was performed to evaluate proliferation.

**Results:**

The cell survival rate was significantly higher in the catheter group. Follow-up examination showed that retinal tack fixation was the only method to maintain the scaffolds attached to host retina for at least 3 weeks, which is the minimal time required for cell integration. Histological staining demonstrated slight glial fibrillary acidic protein (GFAP) accumulation in the retinal tack insertion area.

**Conclusions:**

The established surgical procedure offers a new insight into research of epiretinal cell replacement therapy in rhesus eyes. The successful delivery and long-term fixation provide a prerequisite for cell migration and integration.

## 1. Introduction

Currently, stem cell therapy has been well developed in ophthalmology. The most common approaches for delivering donor cells, including stem cells, retinal progenitors, retinal-pigmented epithelium (RPE), and retinal ganglion cells (RGCs), are subretinal injection [1] and intravitreous injection. These two approaches have been applied to various disease models according to their anatomical and pathological changes. Therefore, in the case of inner retinal diseases, particularly glaucoma, intravitreous injection is the primary choice [2].

However, there was an obvious disadvantage in this approach: targeted delivery and transplantation could not be achieved. The cells were injected in suspension and naturally diffused along the vitreous cavity. To minimize the diffuse distribution, researchers separated the vitreous cavity from the retina [3]; however, a thin cluster along the epiretinal membrane was formed at 2 days after surgery. At a later time point, the transplanted cell suspension spread along the vitreous cavity, attaching to the lens capsule. Moreover, due to the large crystalline lens and small volume of the vitreous cavity, intravitreally injected donor cells migrated for much shorter distances to the inner retinal surface in the rodent eye. In animals with large vitreous cavities, such as felines, the pronounced aggregation of the transplanted cells was observed after injection [4]. Additionally, the inner limiting membrane acts as a barrier blocking migration [5]. Even worse, primates have a more impenetrable ILM than rodents, except in and around the fovea [6]. To solve this problem, a scaffold for stem cells to temporarily adhere and subsequently migrate into the host retina in a specific site is needed [4, 7]. In addition, a specific device for the targeted delivery of the scaffold is also required.

However, whether the transplanted cells could migrate and connect to the host retina is another main problem. Fortunately, significant advances have recently been made towards RGCs/RGC precursors replacement therapies in small animals. Venugopalan and colleagues transplanted mice RGCs into uninjured adult rat retinas [8]. The transplanted RGCs migrated into the host ganglion cell layer, extending neuritis into the inner plexiform layer, terminating in the geniculate nucleus and the superior colliculus. Further, Lim et al. showed that the regenerated RGC axons were capable of finding their own pathway to the specific target in brains according to the RGC type [9]. However, in rhesus monkeys, the precondition of cell migration and reconnection was to provide sufficient contact time for donor cells with the host retina. Thus, the cellular scaffold should be fixed on the host retina for an adequate time.

The rhesus macaque is a well-known system in medicine. Due to its close anatomical and physiological relation to humans, rhesus macaques have been extensively used in medical and biological research on humans. In the aspect of eye research, monkeys are outstanding for their retinal and optic nerve anatomy, which are almost identical to humans. As a glaucoma model, monkeys have close phylogeny and high homology with humans [10]. Therefore, rhesus macaques with chronic glaucoma represent a promising model for the future transplantation of stem cell-combined scaffolds.

The purpose of the present study was to establish a surgical procedure to accurately deliver and fix the cellular scaffolds to facilitate stem cell migration to the inner retinas of rhesus monkeys.

## 2. Materials and Methods

### 2.1. Retinal Progenitors and PLGA Composite

**Figure 1:**
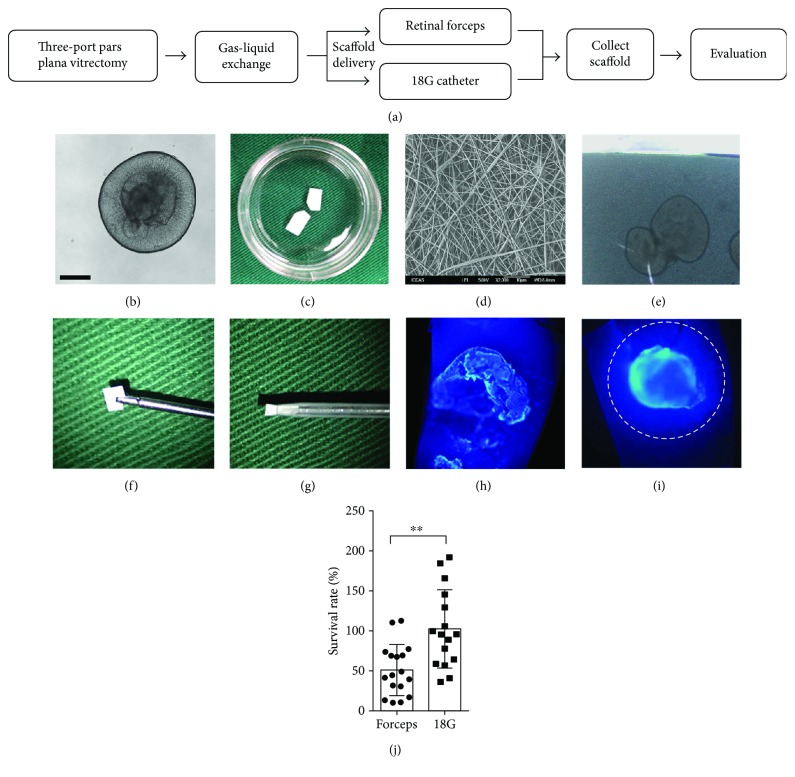
Comparison between the two PLGA scaffold delivery methods. (a) Surgical procedures with the two different delivery methods. After gas-liquid exchange, cellular scaffolds were delivered either by retinal forceps or 18G catheter. (b) Three-dimensional retinal tissue cultured in suspension (scale bar = 200 *μ*m). (c) The degradable PLGA scaffold in a dish. The size of scaffold is 2 mm ^∗^ 4–8 mm, and one corner was cut to label the cellular side. (d) Scanning electron micrographs of the nanofibers in the PLGA scaffold, which was prepared by electrospinning. (e) Retinal tissue with progenitors was seeded and cultured onto the PLGA scaffold in dish. A few days later, the progenitors migrated and spread out from tissue and were directed towards an RGC fate. (f) Cellular PLGA scaffold delivery by retinal forceps. The scaffold was rolled up edge to edge to wrap and protect the retinal progenitors from scraping during delivering. (g) The half-loaded PLGA scaffold in an 18G catheter. After retracting the core, the scaffold was rolled up and loaded into the catheter. (h) DAPI staining of the cellular scaffold delivered by retinal forceps showed that the retinal tissue was obviously damaged. (i) DAPI staining of the cellular scaffold delivered by 18G catheter demonstrated retinal tissue with intact 3D structure. The dashed circle demonstrates the differentiating and migrating progenitors on surface of scaffold. (j) Comparison of the cell survival rates between the two delivery methods (^∗∗^ *P* < 0.01).

HiPSC cell line: SB line (Cellapy, CA4002106). The cells were maintained in mTeSR™1 medium (STEMCELL Technologies 05850) at 37°C in the presence of 5% CO_2_ and were induced to differentiate into three-dimensional retinal tissue following previous protocols [11]. Briefly, iPS cells were induced to form embryoid bodies (EBs) and sequentially differentiated in N2 (Gibco, 17502-048) and B27 (Gibco, 12587-010) supplements (Figure 1(b)).

The PLGA scaffolds used in the present study were prepared by electrospinning and hydrophilic treatment (Figure 1(c)). The molecular weight was designed as 35,000 to meet the requirement of complete degradation in two months. The pores were formed by nanofibers (Figure 1(d)), providing space for cell adherence and migration. Subsequently, three-dimensional retinal tissue with retinal progenitors was seeded and adhered onto Matrigel-coated (Corning, 354277) PLGA scaffolds (approximately 2 mm × 4 mm × 0.1 mm). Then, the progenitors and PLGA composite were maintained in retinal differentiation medium (supplemented with B27 and 10% FBS (Gibco, 10099-141)) for another five days under 37°C in the presence of 5% CO_2_ until transplantation (Figure 1(e)).

### 2.2. Experimental Animals

The present study was conducted in accordance with the Association Research in Vision and Ophthalmology (ARVO) Statement for the Use of Animals in Ophthalmic and Vision Research. All animal experiments were conducted with the approval of the Animal Research Committee, Zhongshan Ophthalmic Center, Sun Yat-sen University. The rabbits and monkeys were housed in rooms under standard laboratory conditions (18–23°C, 40%–65% humidity, 12 hr light/12 hr dark cycle), with food and water available ad libitum. After surgery, the rabbits and monkeys were cared for by a veterinarian until waking. Tobramycin and dexamethasone eye ointment (Tobradex, Alcon, USA) were administered twice a day until the 30th day after surgery.

Fifty male New Zealand White rabbits (50 eyes) aged twelve weeks were used for composite delivery experiments and randomly assigned to two groups, including the forceps group and the catheter injector group. Six male rhesus monkeys (six eyes) aged four years were used in the composite epiretinal fixation experiments. Initially, three monkeys were randomly subjected to three different surgeries, including gas-liquid exchange, hydrogel adhesion, and retinal tack fixation. One month after surgery, we evaluated the fixation outcome to determine the most suitable surgery and performed the selected surgery three more times in three other monkeys to further confirm the efficiency of the fixation method (with one of the monkey supplied with intravitreous injection of 20 *μ*g/ml rapamycin every week).

### 2.3. Retinal Progenitors and PLGA Composite Delivery Surgery and Evaluation

#### 2.3.1. Preoperative Care and Anesthesia

Dicynone (5 mg/kg) was administered to the rabbits by intramuscular injection at 30 minutes prior to surgery, to decrease bleeding. Anesthesia is induced by the intramuscular injection of 3% sodium pentobarbital (0.5 ml/kg), diazepam (0.5 mg/kg), and Su-Mian-Xin II (0.1 ml/kg, xylazine hydrochloride, Shengda Animal Drug Company, Dunhua, China). In addition, tropicamide eye drops (Mydrin, Alcon, USA) and retrobulbar anesthesia (2% lidocaine, 1-2 ml) are administered after general anesthesia is successfully induced. Adequate anesthesia can last for up to 90 minutes by this program. The animals are placed in a supine position with the cornea paralleling to the operating table. The periocular hair was removed, and the periocular skin, ocular surface, and conjunctival sac were disinfected with 5% povidone iodine.

#### 2.3.2. Delivering Scaffold

Standard 23G pars plana vitrectomy (cut rate 0–1500; aspiration 100–300) and gas-liquid exchange (Accurus, Alcon, USA) would be performed prior to the transplantation of the cellular scaffolds. Subsequently, the incision was enlarged to 18G to provide adequate space for scaffolds delivery. To deliver the cellular scaffold into the vitreous cavity through a minimally invasive incision, two methods were developed: the scaffold was grasped by retinal forceps or injected by 18G catheter (Figure 1(a)). For forceps grasping, the cellular scaffold was rolled up with the progenitor side inward and grasped at the edge (Figure 1(f)). The scaffolds were delivered into the vitreous cavity through the 18G scleral incision. For catheter injection, the PLGA scaffold was carefully loaded into the tip of an 18G catheter (modified from 18G venous catheter, BD Intima II™ REF 383405) with forceps, after retracting the core (Figure 1(g)). Subsequently, the scaffolds were injected into the vitreous cavity through an 18G scleral incision by pushing the core. To compare the efficiency of these two methods in protecting the adherent cells on scaffolds, fifty cellular scaffolds were divided into two groups and delivered into rabbit eyes by either grasping or injection.

#### 2.3.3. Cell Loss Assay

After surgery, the scaffolds were carefully removed from the vitreous cavity through an enlarged incision to prevent from any injury to the cells on the scaffold. Cell Counting Kit-8 (Dojindo, Japan) was used to detect the cell numbers on the scaffold prior to and after surgery. The cellular scaffolds were settled in 96-well plates cultured in DMEM (without phenol red, Gibco 21063-029) with 10% FBS (Gibco, 10099-141). The reagent was added to the wells, and the number of cells on the scaffold was measured by the absorbance (450 nm) of reduced water-soluble tetrazolium salt (WST) at the indicated time points.

#### 2.3.4. DAPI Staining

The cellular scaffolds were fixed in 4% paraformaldehyde (PFA, Sigma) for 30 minutes. DAPI (4′,6-diamidino-2-phenylindole) was used for nuclear counterstaining (Molecular Probes). Fluorescence images were acquired with a fluorescence microscope (Olympus).

### 2.4. PLGA Scaffold Epiretinal Fixation Surgery and Evaluation

#### 2.4.1. Fixation Surgery

**Figure 2:**
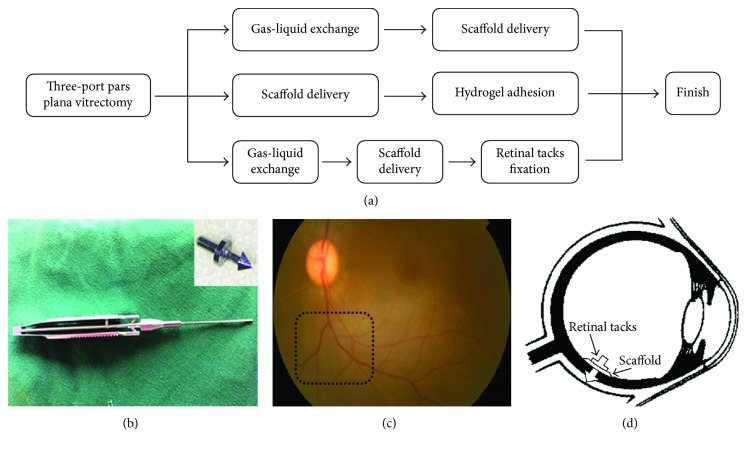
The procedures and equipment for implantation surgery. (a) The surgical procedures of the three different scaffold fixation methods included adhesion after gas-liquid exchange, tamponaded by hydrogel, and fixed by retinal tacks. (b) Retinal tacking and forceps for the tack insertion. (c) The preset site for the PLGA scaffold fixation. (d) Schematic diagram of the PLGA scaffold fixation by retinal tacks.

The perioperative management of scaffold epiretinal fixation surgery in monkeys is the same as scaffold delivery surgery. We compared three common methods to fix the epiretinal implant: attached to the host retina after gas-liquid exchange, tamponaded by hydrogel, and fixed by retinal tack [1]. Gas-liquid exchange: after vitrectomy, gas-liquid exchange was performed, and the scaffolds were delivered into vitreous cavity. Retinal forceps were used to adjust the scaffold position of the epiretinal attachment, and subsequently, the scleral and conjunctiva incisions were sutured [2]. Hydrogel (Laysan Bio Inc.): after vitrectomy and scaffold delivery, 0.5 ml hydrogel was injected into the vitreous cavity over the scaffold by a syringe with 25G needle to push scaffold adhere to the retina [3]. Retinal tacking: after vitrectomy, gas-liquid exchange, and scaffold delivery, two retinal tacks were inserted (Geuder GmbH, Heidelberg, Germany) to fix the scaffold onto the retina (Figure 2).

We evaluated the position of the scaffold and surgery complications by fundus camera (TRC-50DX, Topcon, Japan) and spectral domain optical coherence tomography (Spectralis OCT, Heidelberg, Germany) at 1, 7, and 30 days after surgery.

#### 2.4.2. Immunofluorescence and Histology

On the 30th day, the rhesus globes with retinal tacks were collected for hematoxylin and eosin staining and immunofluorescence detection to evaluate the inflammation and proliferation caused by retinal tacks. The rhesus globes were fixed in 4% paraformaldehyde (PFA, Sigma) overnight. Then, the globes were dissected to maintain 3-4 mm of tissue around the retinal tacks. After embedding, tissue sectioning was performed until reaching the retinal tack. Subsequently, the tack was gently removed by forceps. Immunofluorescence and HE staining were performed as previously described [11, 12]. Antibodies against the following proteins were used at the indicated dilutions: GFAP (mouse, 1 : 300, Cell Signaling Technology, 3670) and iba-1 (rabbit, 1 : 200, Abcam, Ab178680). Secondary antibodies used included the corresponding species-specific Alexa Fluor-555-conjugated antibodies (1 : 500, Gibco). DAPI (4′,6-diamidino-2-phenylindole) was used for nuclear counterstaining (Molecular Probes). Fluorescence images were acquired with an LSM 510 confocal microscope (Zeiss) and a fluorescence microscope (Olympus).

#### 2.4.3. Statistical Analysis

For statistical comparisons, these values were subjected to *t*-test in SPSS Statistics Version 23.0 (SPSS Inc., IBM Company, Armonk, NY, USA) and Prism Software Version 7 (GraphPad Software Inc., La Jolla, CA, USA). The differences were considered statistically significant when the *P* value was less than 0.05.

## 3. Result

### 3.1. Delivery of Retinal Progenitors and PLGA Composite in Rabbits

Immunostaining for postsurgery scaffolds demonstrated that 18G could provide much better protection to the retinal tissue during delivery (Figures 1(h) and 1(i)). Compared to the damage of retinal tissue in the forceps group, the retinal tissue was much better preserved in the 18G catheter group with migrating progenitors. As shown in Table 1 and Figure 1(j), the OD value and cell survival rate were significantly decreased in the retinal forceps group after surgery (*P* < 0.01), while in the 18G catheter group, no statistically significant difference was found between preoperation and postoperation (*P* = 0.863 > 0.05).

### 3.2. Epiretinal Fixation of the PLGA Scaffold in Rhesus Eyes

**Figure 3:**
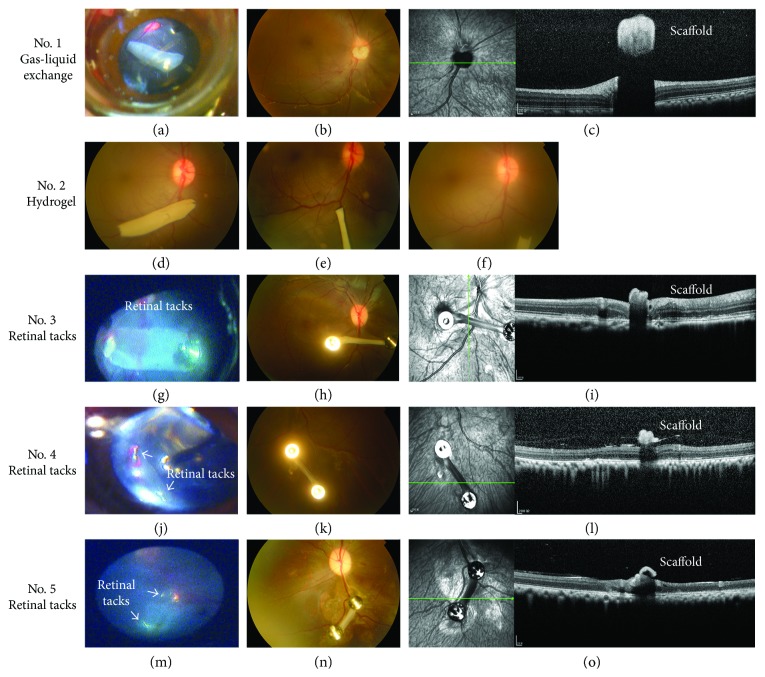
The fundus photograph and OCT scan of PLGA scaffold epiretinal fixation. (a) Photography of PLGA scaffold fixation after gas-liquid exchange in the surgery. (b) Fundus photography at one-month post-PLGA scaffold fixation after gas-liquid exchange. (c) SD-OCT photography at one month after PLGA scaffold fixation after gas-liquid exchange. (d) Photography of PLGA scaffold fixation by hydrogel in the surgery. (e) Fundus photography at one week after PLGA scaffold fixation by hydrogel. (f) Fundus photography at one month after PLGA scaffold fixation by hydrogel. (g, j, and m) Photography of PLGA scaffold fixation by retinal tacks in the surgery. (h, k, and n) Fundus photography at one month after PLGA scaffold fixation by retinal tacks. (i, l, and o) SD-OCT at one month after PLGA scaffold fixation by retinal tacks. The two sides of the scaffold were degraded, and the remaining central region was curled.

Due to the large vitreous cavity of the rhesus eye, one of the main obstacles in epiretinal implantation is the fixation of implants. Three different ways to fix the epiretinal implantation were tested: attached to the host retina after gas-liquid exchange, tamponaded by hydrogel, and fixed by retinal tacks. The results of the present study showed that the scaffold could be adhered to a specific site on retina during the surgery by all three methods (Figure 3). However, only the retinal tacks were able to completely fix the scaffold flat onto the retina, while the edges of the scaffold were curled by gas-liquid exchange only or using hydrogel. Mild to moderate hemorrhage was observed around one of the two retinal tacks after tack insertion but clotted fast (Figures 3(a), 3(d), and 3(g)). On the 7th day postoperation, the scaffold was displaced from the original site in gel-adherence group, but clear fundus photographs were difficult to obtain in the gas-liquid exchange and retinal tack groups because the gas in the globes was not completely absorbed. On the 30th day postoperation, the scaffolds were completely out of position in the gel-adherence and gas-liquid exchange groups. In the gel-adherence group, no scaffold was observed in the preset position. In the gas-liquid exchange group, the scaffold was degraded, curled, and moved from the preset position to the upside of the optic nerve head. Moreover, the scaffold floated with eye movement but did not tightly stick to the optic nerve head. However, the scaffold remained at the preset site and was tightly attached to host retina in the retinal tack group. The two sides of the scaffold were degraded, and the remaining central region was curled into a strip shape (Figures 3(c), 3(f), and 3(i)). Fundus photos and OCT for the monkey treated with intravitreous rapamycin were not shown here. Due to the signal obstruction by PLGA scaffold, the below retina could not be observed in the OCT. In addition, the hemorrhage was completely absorbed, and no retinal edema and detachment was found. No case of endophthalmitis was observed during postoperative follow-up in all the four monkeys (Table 2).

### 3.3. Mild Glial Fibrillary Acidic Protein (GFAP) Accumulation after Retinal Tack Insertion

**Figure 4:**
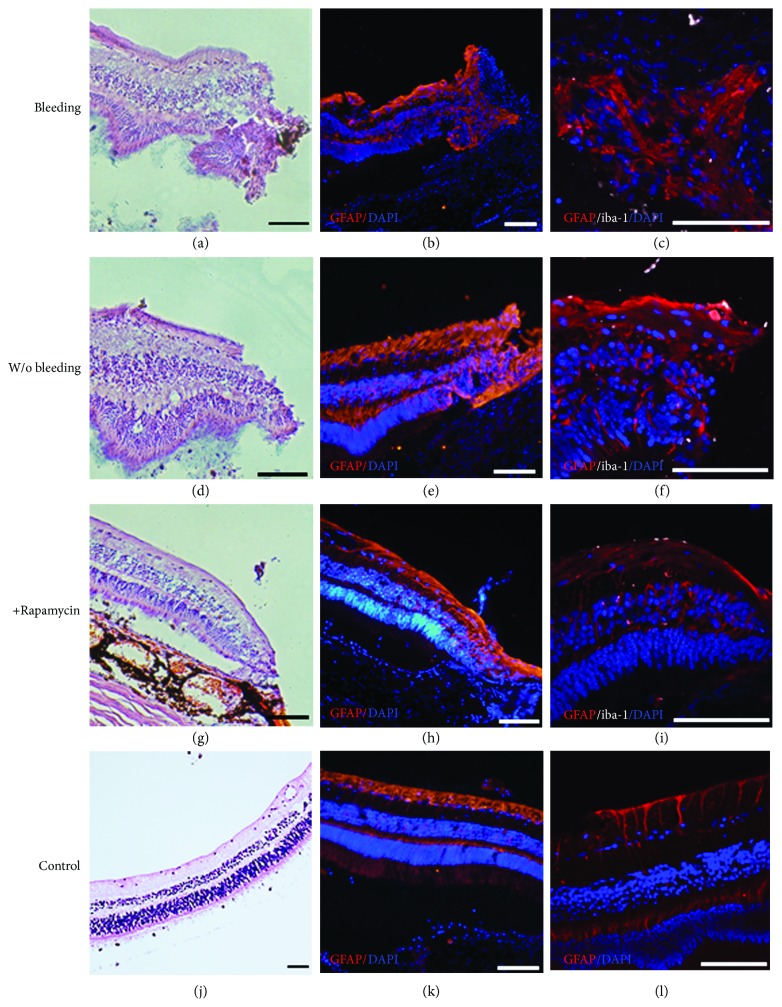
Glial fibrillary acidic protein (GFAP) accumulations around the retinal tack insertion area. Each line demonstrated the HE staining and immunofluorescence at lower and higher magnifications. (a–c) showed retinas with bleeding when inserting the tack. The thickening of the IPL and excessive accumulation of GFAP were observed. (d–f) showed the insertion area of the retina without bleeding during surgery. Only slight GFAP accumulation was observed, and there was no obvious thickening of the inner plexiform layer (IPL). (g–i) showed much slighter retinal inflammation in the presence of rapamycin. (j–l) showed the appearance of a control rhesus retina. Moreover, iba-1 staining showed no activation of microglia (scale bar = 100 *μ*m).

Histological examination by hematoxylin and eosin staining revealed a thickening of the inner plexiform layer around the retinal tack, except the intravitreous rapamycin group (Figures 4(a), 4(d), and 4(g)). In the immunofluorescence assay, mild accumulations of GFAP were observed in most cases. An obvious thickening of the inner plexiform layer (IPL) occurred in the case of moderately bleeding retinal tacks, consistent with the HE test. GFAP staining also showed more excessive accumulation in this case (Figures 4(b) and 4(c)). Additionally, the examination of retina without obvious bleeding demonstrated relatively slight GFAP accumulation (Figures 4(e) and 4(f)) compared to the control retina (Figures 4(k) and 4(l)). Iba-1 staining also showed that obvious microglial activation was not found. Moreover, in the presence of immunosuppressor, retinal inflammation was even more obviously reduced.

### 3.4. The Main Steps of Implantation Surgery in Rhesus Retinas

**Figure 5:**
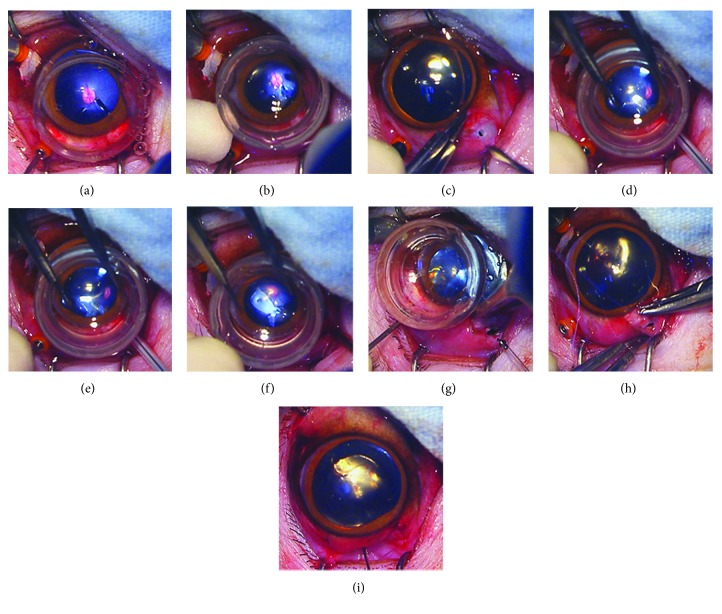
The procedure for rhesus epiretinal implantation. (a) General 23G vitrectomy. (b) Gas-liquid exchange. (c) Sclerotomy enlarging. (d, e) Injecting the epiretinal implant. (f) Flattening the implant with the progenitor side against the host retina. (g) Retinal tack insertion. (h) Closing the scleral and conjunctival incision. (i) Postoperative antibiotics application.

The main steps of the stem cellular scaffold epiretinal implantation surgery were established as follows: after a standard 23G pars plana vitrectomy (Figure 5(a)) and gas-liquid exchange (Figure 5(b)) were performed, a cellular scaffold was injected through an 18G catheter under direct vision and flattened with the progenitor side against inner retinal surface (Figures 5(d)–5(f)). Then, the implant was fixed by two retinal tacks (Figures 5(g)–5(i)).

## 4. Discussion

Intraocular transplantation of stem cells or retinal progenitor has long been a popular field in ophthalmological regenerative medicine. Subretinal injection is the most widely used method in delivering donor cells. However, for inner retinal diseases, epiretinal implantation might be a better choice. Notably, studies on epiretinal stem cell translation are rare. Three main obstacles need resolution: effective delivery of donor cells/tissues, long-term contact with host retina to facilitate migration, and applicability in animal models with large vitreous cavities.

Recent studies on iPS-derived ganglion cell transplantation showed that the cells survived and integrated into the eyes of mice, which provided a promising approach to treat glaucoma [8, 13]. However, these studies were performed in small animal models, of which the anatomical structure and surgical methods possessed low resemblance to those for human eyes. The present study provided an approach to cellular epiretinal tissue-engineering scaffold implantation in rhesus eyes, which successfully delivered and fixed the scaffold on the surface of the retina in relatively large vitreous cavities. Specifically, the present transplantation surgery was more targeted and precise than intravitreous injection, less invasive than prosthesis implantation, and facilitated better scaffold fixation than the reported feline surgery [14].

The suitable delivery methods were evaluated at the first part, because it is always one of the most important things in the transplantation surgery to minimize the lesion of donor retinal progenitors. Considering the sample size needed for delivery comparison and the high cost of rhesus monkeys, rabbits were used in the first part of the present study. In addition, the size of the rabbit eyeballs was similar to those of primates, making it easy to perform three-port pars plana vitrectomy. The scaffold delivery by catheter was far more effective than that by retinal forceps in the present study. The venous catheter, from which the deliver catheter was modified, is a common medical tool, which is low cost and easy to obtain, with an advantage for protecting cells in the lumen, making this method a suitable candidate for cellular scaffold delivery in transplantation surgery.

Secondly, to reduce the surgical lesion in host is also critical in the procedures of transplantation. The most common epiretinal implantation was retinal prosthesis, including Argus II, EPIRET3, IMI, and IRIS [15]. For example, the general surgical procedure of Argus II array implantation was to perform a 3-port pars plana vitrectomy, followed by array insertion through a 5.2 mm sclerotomy and retinal tack fixation [16]. However, the procedure was too invasive to perform in a nonblind eye. In glaucoma patients, stem cell therapy was not only the last choice in the late stage but also a hopeful way of controlling the vision loss in earlier stages. Thus, the surgery was supposed to be minimally invasive. Moreover, compared to another study on scaffold epiretinal implantation in felines [14], the present approach did not include lensectomy. Consequently, the total invasion in the present procedure was only two 23G incisions and an 18G sclerotomy, which were almost the same as a general vitrectomy.

After vitrectomy, the fixation was another problem in large vitreous cavity transplantation because there is no chance for cell migration if the cellular scaffold floats in the vitreous cavity. Studies have shown that the scaffold was simply attached to the inner surface of retina after gas-liquid exchange [14]. Unfortunately, according to the present observation, the scaffold would not remain in the preset position after the gas was absorbed. Concerning the invasion of retinal tacks, bio-glues may be a noninvasive method for scaffold fixation. In consideration of its safety, relative strength of adhesion, and consistency, SS-PEG hydrogel is a candidate for use as an adhesive in scaffold fixation [17]. However, hydrogel was not able to fix the scaffold for more than one week; according to a previous study [8], transplanted neurons migrated to the inner plexiform layer of the host retina after more than one week and formed synaptic puncta after one month. Finally, only retinal tacks could fix the scaffold in the preset site until it was completely degraded, without any movement. The fixation problem was confirmedly solved by retinal tacks. Moreover, retinal tack insertion did disturb host retinal structure, as shown in histological examinations. This was the reason why retinal tack insertion was gradually replaced by silicon oil tamponade or laser photocoagulation in managing retinal tears. However, gas tamponade, hydrogel adherence, nor laser photocoagulation could fix the scaffold for enough time, while silicon oil would be against cell growth. As the only reliable method in stem cell scaffold fixation, retinal tack would still be an acceptable choice, for the benefits outweigh the risks. Additionally, in the foreseeable future, the clinical stem cell transplantation research will performed firstly in primate models and then in absolute glaucoma patients. Thus, risks caused by retinal tack insertion were not unacceptable at present stage.

On the other hand, retinal tacks insertion may lead to concerns about complications such as bleeding and glial cell proliferation. In fact, the safety of retinal tacking had previously been clinically confirmed in retinal detachment repair surgery, including follow-up cases for ten years [18]. According to our results, no retinal detachment was observed after our surgery. Only mild hemorrhage was observed in the surgery, and the mild bleeding was absorbed in one week. Moreover, bleeding could be prevented by avoiding the main blood vessel upon insertion and by improving the surgical skills.

Another concern would be that mechanical injuries were triggers of glial cell proliferation, preventing donor cell migration. GFAP is one of the intermediate filament proteins that react to mechanical injuries to the retina and a highly stable cytoskeletal component in Muller cells. The different expression levels of GFAP may play roles in the formation of glial scars in the retina [19]. Previous studies using domestic pigs showed that the injuries by retinal tacks provoke the up-regulation of glial fibrillary acidic protein (GFAP), which may be associated with the subsequent impairment of the retina [20]. In these studies, the GFAP extensively accumulated in the insertion area. Another previous study showed that the gliosis may impede the migration and integration of transplanted photoreceptors [21]. Additionally, in vitro studies indicated that anti-inflammation treatment including rapamycin not only reduced local inflammation and proliferation, but also provided a protective effect of neurons [22, 23]. Consistently, with weekly intravitreous rapamycin injection, the slight glial reaction induced by retinal tack was notably improved in present surgery. This effect may enable the cells to migrate and integrate with the least interruption by the glial cells.

As the carrier of donor retinal progenitors in this study, PLGA was acknowledged for its nontoxic and well-tolerated effects, with approval for clinical use in humans by the US Food and Drug Administration (FDA) [24]. The present scaffold provided a suitable platform for RGC to differentiate during migration, and at the proper time point, the scaffold could be designed to fully degrade.

Furthermore, the PLGA scaffold could also perform as a carrier for the controlled release system of growth factors or immunosuppressors which shows a prospective application in stem cell-based transplantation field. In later research, less invasive procedure must be investigated, since the stem cell therapy may also be used to treat eye diseases in earlier stages, to protect visual function before loss. Therefore, further researches in minimally invasive method of scaffold fixation, for example, new degradable tacks would be promising.

## 5. Conclusions

In summary, the present study is the first report of the epiretinal implantation surgical procedure in rhesus eyes for the delivery and epiretinal fixation of a degradable scaffold with hiPSC-derived retinal progenitors. This procedure facilitated the effective and precise stem cell transplantation to inner retina in primates, even in human eyes, which may enable the development of novel regenerative therapies for inner retinal diseases, such as glaucoma.

## Figures and Tables

**Table 1:** The cell loss rate of the two delivery methods (CCK-8).

	Optical density value		*P* value
	Pre-op	Post-op	
Forceps	0.58 ± 0.140	0.27 ± 0.164	<0.01
18G catheter	0.59 ± 0.170	0.58 ± 0.283	0.863

**Table 2:** Scaffold fixation outcomes and complications of scaffold fixation surgery with retinal tacks in rhesus eyes.

Number	Surgery	Fixation	Fundus hemorrhage	Retinal detachment	Endophthalmitis
1	Gas-liquid exchange	Failed	None	None	None
2	Hydrogel	Failed	None	None	None
3	Retinal tacks	Success	Mild^∗^	None	None
4	Retinal tacks	Success	Mild^∗^	None	None
5	Retinal tacks	Success	Moderate^∗∗^	None	None
6	Retinal tacks (+rapamycin)	Success	Mild^∗^	None	None

^∗^Mild hemorrhage in one of the two tacks. ^∗∗^Moderate hemorrhage in one of the two tacks.
